# The StrongerMemory Program: A Brain Health Intervention for Mild Cognitive Impairment

**DOI:** 10.1093/geroni/igab046.552

**Published:** 2021-12-17

**Authors:** Catherine Tompkins, Emily Ihara, Rob Liebreich, Jessica Fredericksen, Lauren Bradley

**Affiliations:** 1 George Mason University, Fairfax, Virginia, United States; 2 Goodwin House, Falls Church, Virginia, United States

## Abstract

The StrongerMemory program is a brain health intervention targeting older adults with mild cognitive impairment (MCI). Approximately 20% of individuals over 65 years old have MCI and 38% will potentially develop dementia after 5 years of MCI onset. Participants in the 16-week StrongerMemory program practice exercises involving math, writing, and reading aloud for 20-30 minutes a day, stimulating the prefrontal cortex of the brain, which governs the brain’s ability to retrieve memories. The current research exploring participants’ experiences with the StrongerMemory program was approved by the University’s Institutional Review Board. A semi-structured interview was implemented with each participant and the interviews took about 15 to 30 minutes to complete. There was a total of 24 respondents associated with a local continuing care retirement community, each providing their verbal consent to be interviewed and recorded over Zoom. The respondents were mostly female ranging in age from 65 to 90. Most respondents reported having advanced degrees and were participating in the program on their own, with a spouse or friend. Each interview transcript was coded by at least two members of the Mason research team. Participants reported being motivated to participate in the program due to fearing memory loss because of experiences of family members and friends and others wanted to remain “cognitively fit.” Codes and themes representing participants’ perceptions and reactions to exercises and their challenges and lessons learned from their participation will be discussed, including participants feelings of being “less foggy” and better able to remember information.

